# Estimation of the Basic Reproduction Number of Novel Influenza A (H1N1) pdm09 in Elementary Schools Using the SIR Model

**DOI:** 10.2174/1874434601711010064

**Published:** 2017-06-29

**Authors:** Daisuke Furushima, Shoko Kawano, Yuko Ohno, Masayuki Kakehashi

**Affiliations:** 1 Department of Mathematical Health Science, Osaka University Graduate School of Medicine, Japan; 2 Institute of Biomedical & Health Sciences, Hiroshima University, Japan

**Keywords:** A/H1N1pdm, Absentee survey, Basic reproductive number, Elementary school student, School health, SIR model

## Abstract

**Background:**

The novel influenza A (H1N1) pdm09 (A/H1N1pdm) pandemic of 2009-2010 had a great impact on society.

**Objective:**

We analyzed data from the absentee survey, conducted in elementary schools of Oita City, to evaluate the A/H1N1pdm pandemic and to estimate the basic reproductive number (R_0 _) of this novel strain.

**Method:**

We summarized the overall absentee data and calculated the cumulative infection rate. Then, we classified the data into 3 groups according to school size: small (<300 students), medium (300–600 students), and large (>600 students). Last, we estimated the R_0 _ value by using the Susceptible-Infected-Recovered (SIR) mathematical model.

**Results:**

Data from 60 schools and 27,403 students were analyzed. The overall cumulative infection rate was 44.4%. There were no significant differences among the grades, but the cumulative infection rate increased as the school size increased, being 37.7%, 44.4%, and 46.6% in the small, medium, and large school groups, respectively. The optimal R_0 _ value was 1.33, comparable with that previously reported. The data from the absentee survey were reliable, with no missing values. Hence, the R_0 _ derived from the SIR model closely reflected the observed R_0 _. The findings support previous reports that school children are most susceptible to A/H1N1pdm virus infection and suggest that the scale of an outbreak is associated with the size of the school.

**Conclusion:**

Our results provide further information about the A/H1N1pdm pandemic. We propose that an absentee survey should be implemented in the early stages of an epidemic, to prevent a pandemic.

## 1 INTRODUCTION

In the spring of 2009, after the first case was reported in Mexico, infection with a novel influenza A (H1N1) pdm09 (A/H1N1pdm) virus spread rapidly worldwide [1]. The World Health Organization (WHO) recognized A/H1N1pdm as a global public health emergency on April 25, 2009, and the global influenza pandemic alert level was moved to phase 6 on June 11, 2009 [2]. Although the virulence was lower than that of other pandemics of the 20^th^ century, the A/H1N1pdm In pandemic had a great impact on society, causing confusion [3-6].

In Japan, the first domestic outbreak of A/H1N1pdm was reported on May 16, 2009 in Kobe City in western Japan [7]. After being reported, A/H1N1pdm spread throughout all prefectures; approximately 20 million people were infected and 198 died [8, 9]. According to reports, the number of infected cases increased from August 2009 and peaked in November 2009. The infection was spread mainly among young adults and school children [6, 10]. During the pandemic, surveillance for A/H1N1pdm was implemented throughout Japan. In the initial stage, from April 28 to July 23, 2009, A/H1N1pdm was designated as a case-based reportable disease, and all cases were monitored. Subsequently, it has been monitored through sentinel surveillance as part of the National Epidemiological Surveillance of Infectious Diseases (NESID) program, organized by the Ministry of Health, Labour and Welfare.

The guidelines of the NESID are described elsewhere [11, 12]. Briefly, approximately 10% of all medical institutions are designated sentinel sites. In total, there are approximately 5000 sentinel sites (~3000 pediatric and ~2000 internal medicine institutions). These were determined in relation to the population size of each regional health center’s administrative sector throughout Japan. Each week, the number of influenza cases was reported to the regional health centers and to the Ministry of Health, Labour and Welfare from each sentinel medical institution, using an internet network system. However, the NESID system, because of weekly reporting, had a delay from diagnosis to reporting of at least one week, and, being a national system, it was difficult to identify an outbreak at a local level. Therefore, the Oita City Board of Education implemented a school-based absentee survey (absentee survey) for all public elementary schools in Oita City. The information from the absentee survey was widely used for rapid implementation of infection control measures, including decision-making around the implementation of school closure or school events; however, a detailed analysis of the outbreak was not performed.

Therefore, in this study, we first aimed to evaluate the A/H1N1pdm pandemic among elementary school students in Oita City using the absentee survey data. Then, we aimed to estimate the basic reproduction number (R_0 _), the key parameter quantifying the transmission of a pathogen, using the mathematical technique of the Susceptible–Infected–Recovered (SIR) model to evaluate the rate of infection among elementary school students in Oita City. Our study may contribute toward public health policy for formulating preventive measures against future pandemics of a novel influenza virus strain, and highlights the important role of the SIR model in a pandemic situation.

## 2 MATERIALS AND METHODS

### 2.1 The Absentee Survey Data

Oita City is located in Oita prefecture in the Kyushu area of western Japan. It has a population of approximately 450,000 people. Oita City has only public elementary schools, and approximately 96% of students attending school live in Oita city [13]. An absentee survey was conducted by the Oita City Board of Education between August 1, 2009 and March 31, 2010. In this survey, every public elementary school was obligated to report the daily numbers of absentees due to A/H1N1pdm to the Oita City Board of Education. The parent(s) of each absent student confirmed whether their child had A/H1N1pdm infection; almost all cases had been diagnosed by physicians, using rapid diagnostic tests. The Oita City Board of Education entered absentee data in a secondary, open-source database. The data included the daily number of absent students, an anonymous school identification number, school grade (first to sixth grade; 7–12-year-old children), and the number of enrolled students. In the present study, we treated absent students as infected students.

### 2.2 The SIR model

In this study, we used the basic SIR model framework to estimate the basic reproduction number, denoted as R_0 _, of A/H1N1pdm in the elementary schools of Oita City. The R_0 _ is the key parameter to quantify the transmission power of a pathogen, and is defined as the average number of secondary infections caused by a single infective individual in a completely susceptible population [14]. The SIR model is a mathematical technique that is widely used for prediction of infectious epidemics. The detailed theory of the SIR model has been described elsewhere [15, 16].

The SIR model separates the population into three components, susceptible (S), infected (I), and recovered (R) individuals; people can move through the components from *S* to *I* to *R*. In this study, the model was represented by the following set of three differential equations:

(1) $$\frac{\mathrm{d}S\left(t\right)}{\mathrm{d}t}=\&minus;\beta \left(\frac{S\left(t\right)}{N}\right)I\left(t\right)$$

(2) $$\frac{\mathrm{d}I\left(t\right)}{\mathrm{d}t}=\beta \left(\frac{S\left(t\right)}{N}\right)I\left(t\right)-\gamma I\left(t\right)$$

(3) $$\frac{\mathrm{d}R\left(t\right)}{\mathrm{d}t}=\gamma I\left(t\right)$$

Where, (*t*) *=* unit of time, *β * = basic reproduction number (R_0 _), *γ* = recovery rate, 1/γ = the average infectious period. The population size was conserved in this model, *N = S*(t) + *I*(t) + *R*(t). 

The number of newly infected people from one infected individual per week is given by $R_{0}\frac{S\left(t\right)}{N}$.

As the R_0 _ value was derived from a population made up of both susceptible and non-susceptible people, the population was discounted by the non-susceptible fraction of the population. For instance, if the R_0 _ value was 4 and half of the population had become immune, the newly infected people were calculated as 4 × 0.5 = 2. Therefore, under these circumstances a single case would produce 2 new secondary cases.

### 2.3 Data Processing and Statistical Analysis

We transformed the absentee survey data into week units to address weekend bias, and included data only from September 1, 2009 to exclude the effect of the summer vacation. The infected cases during August were considered as recovered cases by September 1.

First, we summarized the overall absentee data and calculated the cumulative infection rate. Then, we classified the data into 3 different groups according to the size of the schools: small-scale schools (small-scale group)-schools with <300 enrolled students; medium-scale schools (medium-scale group)-schools with 300–600 enrolled students; and large-scale schools (large-scale group)-schools with >600 enrolled students. Next, we estimated the R_0 _ value using the SIR model. We distributed the absentee survey data into 3 components by week unit, as follows: *S*(t), *I*(t), *R*(t). The value of the first week: *S*(t_ 0_), *I*(t_ 0_), *R*(t_ 0_) was set as the initial value of the model. The students infected before September were added into *R*(t_ 0_). The parameter *γ* was fixed at 1.0; according to the model, infected students recovered after one week. The SIR model was rerun, changing the parameter R_0 _ from 1.00 to 1.50 in increments of 0.01, and the best model was selected based on comparison of the observed cumulative infection rate and that of the model. Furthermore, we divided the absentee survey data into 3 periods from survey initiation-12 weeks, 24 weeks, and 30 weeks-to evaluate whether R_0 _ could predict an outbreak from initial observation data. The R_0 _ of each period was estimated using a similar method, and the results were compared.

Statistical analyses were performed using R environment version 3.2.3 (https://www.r-project.org/). Statistical significance was defined as *p* <0.05. The SIR models were created using the S^4^ Simulation System (NTT DATA Mathematical Systems Inc.). We followed the ethical guidelines for epidemiologic research performed in Japan throughout study.

## 3 RESULTS

The overview of the absentee survey is shown in Table (**1**). During this period, 60 schools and 27,403 students (small-scale group: 19 schools and 2,518 students; medium-scale group: 22 schools and 9,911 students; large-scale group: 19 schools and 14,974 students) were enrolled in the absentee survey. There were no deaths due to any cause, including A/H1N1pdm. The number of infected students (cumulative infection rate) was 12,313 (44.9%) overall, with 934 (37.1%) in the small-scale group, 4,401 (44.4%) in the middle-scale group, and 6,978 (46.6%) in the large-scale group. The cumulative rate of infection varied according to a school size, but there were no clear differences in cumulative infection rate according to grade.

The transition of infected students is shown in Fig. (**1**). The number of infected students increased gradually from the end of September 2009 and formed multiple peaks. The first peak was in the week of October 26 to November 1, 2009, and the second peak was in the week of November 23 to 29, 2009.

The initial values of each SIR model are shown in Table (**1**). Eighty-six students were infected before September 2009 (small-scale group: 3, medium-scale group: 28, large-scale group: 55); they were added to. The SIR model best fitted to the actual epidemic transition is shown in Fig. (**2**). The R_0 _ values of the best-fitted model and difference between the cumulative infection rates of the absentee survey data were 1.33 and 0.4%, respectively, overall (model *vs.* absentee survey: 45.3% *vs* 44.9%); 1.26 and 0.3%, respectively, for the small-scale group (36.8% *vs.* 37.1%); 1.32 and 0.1%, respectively for the medium-scale group (44.3% *vs.* 44.4%); and 1.34 and 0.2%, respectively, for the large-scale group (46.4% *vs.* 46.6%). The larger schools had higher R_0 _ values. There were no significant differences between the model and observation except in component *I* of the small-scale group: The p-values related to components *S*, *I*, and *R* were 0.78, 0.02, and 0.73, respectively. The predictive precision of the model for the small-scale group was not high compared with that of the other groups Fig. (**2b**). The estimated transition curve was more gradual than the observations, presenting a difference in trend from the 44^th^ week of 2009.

The optimal R_0 _ value and the predicted cumulative infection rates of the 3 periods are shown in Table (**2**). Except for the small-scale group, the estimated R_0 _ values for the 12-week period and the 24-week period were similar to the R_0 _ values for the overall period (30 weeks); the maximum difference was 0.03. Furthermore, the difference between the predicted and observed cumulative infection rates for each period was < 3.0%. However, the predictive precision was confirmed to be lower in the small-scale group than in other groups; the R_0 _ and cumulative infection rates were overestimated for the 12- and 24-week periods.

## DISCUSSION

The aim of this study was to evaluate the 2009/2010 influenza A/H1N1pdm pandemic among elementary school students in Oita City. To achieve this goal, we analyzed data from the absentee survey that was conducted in all public elementary schools in Oita City. These data were reliable, with no missing values. Moreover, almost all cases had been diagnosed by physicians, using rapid diagnostic tests. From the analysis, we found that the cumulative infection rate among public elementary schools in Oita City was high (44.9%) between August 2009 and March 2010. This finding supports previous epidemiologic reports stating that those most susceptible to infection with the A/H1N1pdm virus were school children, and that the estimated rate of infection among Japanese children aged 5-14 years was 48.4% [6].

Furthermore, we found that the cumulative infection rate showed no notable difference between grades, but differed by school size, being higher in larger schools (37.7%, 44.4%, and 46.6% in the small-scale, medium-scale, and large-scale groups, respectively). This observation suggests that the scale of an outbreak is associated with the size of the school. Differences in exposure (contiguity/contact situation or density of students at school) among students might account for this observation; the mean number of students per classroom was 20.5 (range, 3-41) in small-scale schools and 32.3 (range, 23-41) in large-scale schools. From this result, we suggest that it is necessary to adapt infection control measures according to the size of the school. Further, analysis of the absentee survey data has given much important information regarding this novel influenza pandemic; these data could be used for deciding about infection control measures, such as school closure or postponement of school events. We thus propose that an absentee survey should be implemented in the early stages of an epidemic.

Next, we estimated the R_0 _ value among elementary schools in Oita City using the SIR model.

We changed the R_0 _ values of the SIR model from 1.00 to 1.50, and selected the optimal R_0 _ value by comparing the cumulative infection rate between the model and observation. The optimal R_0 _ value was estimated at 1.33 for elementary schools overall in Oita City. The optimal R_0 _ values for small-scale, medium-scale, and large-scale schools were 1.26, 1.33, and 1.35, respectively.

To consider whether the R_0 _ values could be predictive at the early stage of a pandemic, we estimated R_0 _ for 3 periods: at 12 weeks, 24 weeks, and 30 weeks (overall data) after the first case had presented.

As shown in the results, the optimal R_0 _ value at 12 weeks was not much different from that at 30 weeks (overall data), except for the small-scale group. Moreover, the predicted cumulative infection ratio was almost equivalent to the observation; the difference was < 3.0%. These results suggest that the prediction of a pandemic is possible using data from the early stage of a pandemic. Therefore, it is important to implement surveillance from the beginning of a pandemic. Hence, it is necessary to build an immediately feasible surveillance system for future pandemics of novel influenza.

The results of R_0 _ values estimated in our study were comparable with those reported in previous studies. In a report analyzing the outbreak of A/H1N1pdm in Mexico, the R_0 _ value was estimated to be in the range of 1.4 to 1.6 from epidemiologic analyses, whereas a genetic analysis gave a central estimate of 1.2 [17]. Moreover, based on the influenza-like illness case data from the civilian population, the R_0 _ value was estimated to be in the range from 1.1 to 3.3 [4, 17-19].

Our selected SIR model very closely predicted the actual epidemic situation. However, as shown in the results, the precision in the small-scale group was lower than that of the other groups. The trends of the SIR model were remarkably different after the 44^th^ week of 2009. We hypothesize that the reason for this result is that the contact situation might have affected this outcome; the mean number of students in the classes was 20.5 with a widely dispersed range (3 to 41 students). Therefore, an irregular infection pattern might have occurred in each school, making theoretical prediction difficult. In addition, in this study, we estimated the SIR model by changing only the R_0 _ value; therefore, uncertain elements may not have been considered. The prediction result in smaller sample sizes should be interpreted with caution. In addition, prediction with a more detailed model is needed. However, as a whole, the predictive precision of the SIR model was very high, re-emphasizing that the SIR model is an important method for predicting the epidemic pattern of A/H1N1pdm. In fact, the SIR model was used to evaluate the effectiveness of A/H1N1pdm control and prevention strategies, *i.e.*, the effectiveness of school closure and preventive actions, such as wearing masks or being vaccinated [20, 21]. As common points to all previous studies, the constitution of the model reflects reality, and each parameter is important for high predictive precision. Unrealistic simulation can lead to the implementation of excessive countermeasures and unnecessary civil disorder.

The strengths of our study were that the absentee survey included all public schools in Oita City, the absentee survey data were complete, and almost all of the reported students were diagnosed by physicians, using rapid diagnostic tests. Thus, our estimated results are thought to closely reflect reality. Therefore, the results may contribute to public health policy making. The findings also highlight the important role of SIR modeling in pandemic situations.

However, our study does have several limitations. First, we had not considered the influence of school closures in this study. Several studies have reported on the effect of school closure during the pandemic of A/H1N1pdm, concluding that school closure was an effective intervention for mitigating the spread of A/H1N1pdm and should be implemented for at least 5 days [20, 22, 23]. Therefore, our R_0 _ value may have been underestimated. However, in all elementary schools in Japan, school closure was implemented with constant criteria according to guidelines issued by the Ministry of Health, Labour, and Welfare. Briefly, a class would be terminated when the proportion of infected students in the class reached approximately 10%. Thus, our R_0 _ value was most likely an accurate estimate, even considering the effect of the school closure. Second, because the data were obtained only from public elementary schools in Oita City, generalization of the results is difficult. In particular, the results are likely to be different in settings of intense population migration, such as in urban areas. In urban areas, data from several sources may need to be combined for analysis. Last, our SIR model was simulated under several assumptions. We disregarded contact within families and outside of schools, various preventive behaviors adopted by each student (such as the wearing of masks, hand-washing, gargling), and environmental factors (such as temperature and absolute humidity). Absolute humidity is known to be associated with the onset of infectious disease [24-26]. Hence, a more detailed analysis is desirable to provide a reliable prediction model. However, a complicated model with multiple factors may complicate the analysis and evaluation of the validity of the model.

An advantages of this study was that we estimated the R_ 0_ values using a simple SIR model. In similar studies, the calculation of R_ 0_ was generally complicated, requiring a high level of mathematical knowledge [27-29]. Because the methodology used in the present study allows for easier estimation of R_ 0_, our method can be used by researchers who are not necessarily mathematics experts, *e.g.* researchers in the field of school health.

To conclude, in this study, we used a simple SIR model for estimation; its predictive precision was very high, the reason for this precision is thought to be based on the accuracy of the survey data. The results of this study should help elucidate the pandemic of A/H1N1pdm in 2009 and should provide useful information to prevent future pandemics.

## Figures and Tables

**Fig. (1):**
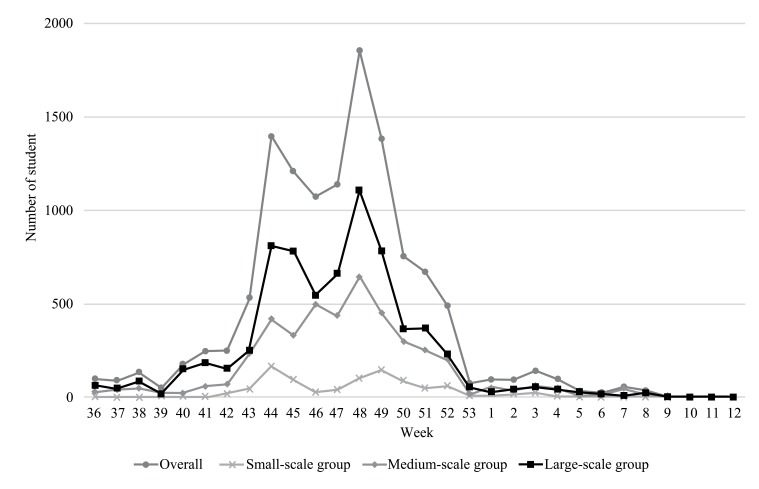
Transition of Oita City elementary school students infected with A/H1N1pdm.

**Fig. (2):**
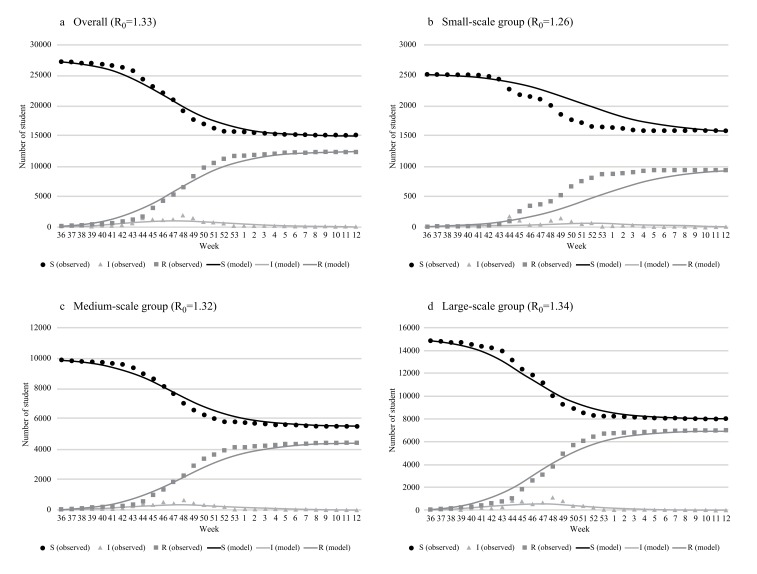
Comparison of the time transition curve of 3 components between observations (absentee survey) and the SIR model.

**Table 1:** Summary of the absentee survey of public elementary school in Oita city.

Variables		Overall (60 schools)	School-scale category		
			Small-scale group (19 schools)	Medium-scale group (22 schools)	Large-scale group (19 schools)
Number of students (%)		27,403 (100.0)	2,518 (100.0)	9,911 (100.0)	14,974 (100.0)
School grade	first	4,415 (16.1)	399 (15.8)	1,596 (16.1)	2,420 (16.2)
	second	4,659 (17.0)	435 (17.3)	1,638 (16.5)	2,586 (17.3)
	third	4,508 (16.5)	383 (15.2)	1,627 (16.4)	2,498 (16.7)
	fourth	4,560 (16.6)	426 (16.9)	1,577 (15.9)	2,557 (17.1)
	fifth	4,585 (16.7)	441 (17.5	1,696 (17.1)	2,448 (16.3)
	sixth	4,676 (17.1)	434 (17.2)	1,777 (17.9)	2,465 (16.5)
Mean number of student in class (range)		29.6 (3 - 41)	20.5 (3 - 41)	29.3 (19 - 40)	32.3 (23 - 41)
Number of infected students (cumulative infected rate)		12,313 (44.9)	934 (37.1)	4,401 (44.4)	6,978 (46.6)
School Grade	first	1,941 (44.0)	141 (35.3)	670 (42.0)	1,130 (46.7)
	second	2,108 (45.2)	169 (38.9)	749 (45.7)	1,190 (46.0)
	third	2,030 (45.0)	119 (31.1)	734 (45.1)	1,177 (47.1)
	fourth	2,027 (44.5)	175 (41.1)	694 (44.0)	1,158 (45.3)
	fifth	2,106 (45.9)	165 (37.4)	764 (45.0)	1,177 (48.1)
	sixth	2,101 (44.9)	165 (38.0)	790 (44.5)	1,146 (46.5)
Initial value of Component S		27,219	2,511	9,852	14,856
Initial value of Component I		98	4	31	63
Initial value of Component R		86	3	28	55

**Table 2:** The result of optimal R_0 _ values and predicted cumulative infected rate for 3 periods.

Data period	Overall		Small-scale group		Medium-scale group		Large-scale group	
	R_0 _	Cumulative infection rate*4	R_0 _	Cumulative infection rate*4	R_0 _	Cumulative infection rate*4	R_0 _	Cumulative infection rate*4
12 week *1	1.31	43.4% (-1.5 %)	1.36	47.6% (10.5 %)	1.32	44.3% (-0.1 %)	1.31	43.6% (3.0 %)
24 week *2	1.33	45.4% (0.4 %)	1.30	41.6% (4.5 % )	1.33	45.2% (0.8 % )	1.35	47.3% (-0.7 % )
30 week *3	1.33	44.4% ( -0.5 % )	1.26	36.8% ( -0.3 % )	1.32	44.3% ( -0.1 % )	1.34	46.4% ( -0.2 % )

*1: Period from 36th week to 47th week (1 Sep 2009 to 22 Nov 2009)

*2: Period from 36th week to 5th week (1 Sep 2009 to 7 Feb 2010)

*3: Period from 36th week to 12th week: overall data (1 Sep 2009 to 28 Mar 2010)

*4: Predicted value in 30 weeks (Parenthesis: difference between observation)
